# Using the intrinsic growth rate of the mosquito population improves spatio-temporal dengue risk estimation

**DOI:** 10.1016/j.actatropica.2020.105519

**Published:** 2020-08

**Authors:** Luigi Sedda, Benjamín M. Taylor, Alvaro E. Eiras, João Trindade Marques, Rod J. Dillon

**Affiliations:** a Lancaster Medical School, Furness Building, Lancaster University, Lancaster, LA1 4YG, UK; b Centre for Health Informatics, Computing, and Statistics (CHICAS), Lancaster Medical School, Furness Building, Lancaster University, Lancaster, LA1 4YG, UK; c Department of Parasitology, Instituto de Ciências Biológicas, Universidade Federal de Minas Gerais, Belo Horizonte, Minas Gerais, CEP 30270-901, Brazil; d Department of Biochemistry and Immunology, Instituto de Ciências Biológicas, Universidade Federal de Minas Gerais, Belo Horizonte, Minas Gerais, CEP 30270-901, Brazil; e Institut de biologie moléculaire et cellulaire, Université de Strasbourg, CNRS UPR9022, Inserm U1257, 67084 Strasbourrg, France; f Biomedical and Life Sciences, Furness Building, Lancaster University, Lancaster, LA1 4YG, UK

**Keywords:** Moran curve, Ricker model, Density dependent and independent mortalities, Log-Gaussian cox process, Dengue, Aedes aegypti

## Abstract

Understanding geographic population dynamics of mosquitoes is an essential requirement for estimating the risk of mosquito-borne disease transmission and geographically targeted interventions. However, the use of population dynamics measures, such as the intrinsic growth rate, as predictors in spatio-temporal point processes has not been investigated before. In this work we compared the predictive accuracy of four spatio-temporal log-Gaussian Cox models: (i) With no predictors; (ii) mosquito abundance as predictor; (iii) intrinsic growth rate as predictor; (iv) intrinsic growth rate and mosquito abundance as predictors. This analysis is based on *Aedes aegypti* mosquito surveillance and human dengue data obtained from the urban area of Caratinga, Brazil. We used a statistical Moran Curve approach to estimate the intrinsic growth rate and a zero inflated Poisson kriging model for estimating mosquito abundance at locations of dengue cases. The incidence of dengue cases was positively associated with mosquito intrinsic growth rate and this model outperformed, in terms of predictive accuracy, the abundance and the null models. The latter includes only the spatio-temporal random effect but no predictors. In the light of these results we suggest that the intrinsic growth rate should be investigated further as a potential tool for predicting the risk of dengue transmission and targeting health interventions for vector-borne diseases.

## Highlights

• Vector indices are often poor predictors of geographic disease risk.
• When longitudinal vector surveillance data is available, vector population dynamics parameters can be estimated.
• Vector population dynamics are investigated as a measure of disease risk.
• In this work we have shown that mosquito intrinsic growth rate has a larger predictive accuracy for dengue incidence than mosquito abundance.
• In the light of this result we encourage vector surveillance and control agencies/scientist to explore the use of intrinsic growth rate as index for disease risk.

## 1 Introduction

Understanding mosquito population dynamics is fundamental to developing models for estimating the entomological risk of mosquito-borne disease transmission that can be used for effective mosquito surveillance and control, e.g. precision public health. Current indexes (e.g. Breteau, number of disease cases surpassing a pre-defined threshold) are efficient only when mosquito infestation is already happening, or when multiple indexes are combined (Vargas et al., 2015). Despite the variety of methods and their availability (Tonnang et al., 2017), current risk assessment models for vector-borne diseases are mainly based on static species occurrence models. Such models use spatialization of presence/absence or count abundance and lack information about the insect population dynamics, i.e. mortality, fertility and density dependence effects (see for example the recommendations from (Ehrlen and Morris 2015)). When longitudinal data is available, dynamic models can provide essential information for vector-borne disease risk management (Cromwell et al., 2017; Tonnang et al., 2017).

The dynamicity of a mosquito population depends on its behaviour (i.e. timing of diapause, host seeking) and the dependence of its demographic parameters on environmental changes. Mosquito populations can respond rapidly (within hours to days) to meteorological changes (Parham et al., 2012; Armbruster 2016), resulting in abrupt variations in population abundance (Jian et al., 2014a), especially in areas where rainfall and temperature are strongly seasonal (Jian et al., 2014b). Vector mortality/survival is one of the population dynamics parameters used to describe disease transmission (Brady et al., 2014). Vector mortality depends on density dependent mortality (DD), which for mosquitoes has been suggested as one of the main parameters affecting vector control measures (Legros et al., 2016) at different insect life stages particularly the aquatic stage (Phuc et al., 2007). DD is modulated by a multiplicity of social and trophic interactions, such as cannibalism, competition, crowding, co-operation, diseases, mutualism, parasitism, parasitoidism, predation, and reproductive behaviour (Gerber et al., 2005; Herrando-Pérez et al., 2012).

The aim of the study was to find out if the spatio-temporal variation in the intrinsic growth rate is better associated with the incidence of dengue cases than spatio-temporal mosquito abundance. The geographic association between dengue cases and mosquito intrinsic growth rate and abundance was evaluated for a dengue endemic area in Brazil. The advantage in the use of intrinsic growth rate is that its estimation only requires abundance data when compared to other indexes (reproductive number, R0, and vectorial capacity (Reiner et al., 2013)).

Dengue is the second most important vector-borne disease worldwide with 2.5 billion people at risk (World Health Organization 2014). Dengue and other arboviral diseases (Zika and chikungunya) lack efficient vaccines, are transmitted by the same *Aedes* mosquito vectors, and their control still relies on preventing contact between mosquito and humans (World Health Organization 2009). The dengue cycle involves mosquitoes and humans, although limited virus circulation in other vertebrate hosts has also been reported (Diallo et al., 2003; de Thoisy, Lacoste et al. 2009). According to the World Health Organization, Brazil currently occupies the first place in the ranking of reported dengue cases in the world with incidence rates increasing since 2004 (Fares et al., 2015) and with poorly understood efficacy of its control (Bowman et al., 2016).

*Aedes aegypti* (L.) (Diptera: Culicidae) mosquito is responsible for the urban transmission of the dengue virus (Kraemer et al., 2015). Its populations are considered to be regulated by a strong density dependent mortality (Robert et al., 2014). *Ae. aegypti* is highly anthropophilic; water containers or drains associated with human habitation are the principal breeding habitat. These mosquitoes lay eggs on the inner wall of water filled containers. Female *Ae. aegypti* feed almost exclusively on humans in daylight hours and typically rest indoors. This mosquito species is very mobile, although the flight range during its life span is no more than 1 km (Sarfraz et al., 2014) and the majority of the population stays within 200 m (Sarfraz et al., 2012). Oviposition and aquatic stages of *Ae. aegypti* are regulated by density dependence (Lana et al., 2014), while adults may be regulated mainly by density independent events, with few cases involving density dependent mortality.

This work is composed of two parts. Firstly, we used a process-based statistical compartmental framework (which assumes independence between some of the population parameters) to estimate the: unlimited growth *λ_0_* (Eq. (3) in Methods); the density independent mortality, *DI* (Eq. (4)); the potential total mortality, *DT* (Eq. (5)); the density dependence parameters, *d* and *α* (Eq. (6)); the density dependence, *DD* (Eq. (8)); and the intrinsic growth rate, *IGR* (Eq. (10)). Secondly, we evaluated whether the intrinsic growth rate is significantly associated with dengue spatio-temporal incidence and how it compares with a mosquito abundance model and a null model (intercept+spatio-temporal random effect). This section of the analysis is based on a spatio-temporal log-Gaussian Cox model (Taylor et al., 2018).

## 2 Materials

### 2.1 Study area

**Fig. 1:**
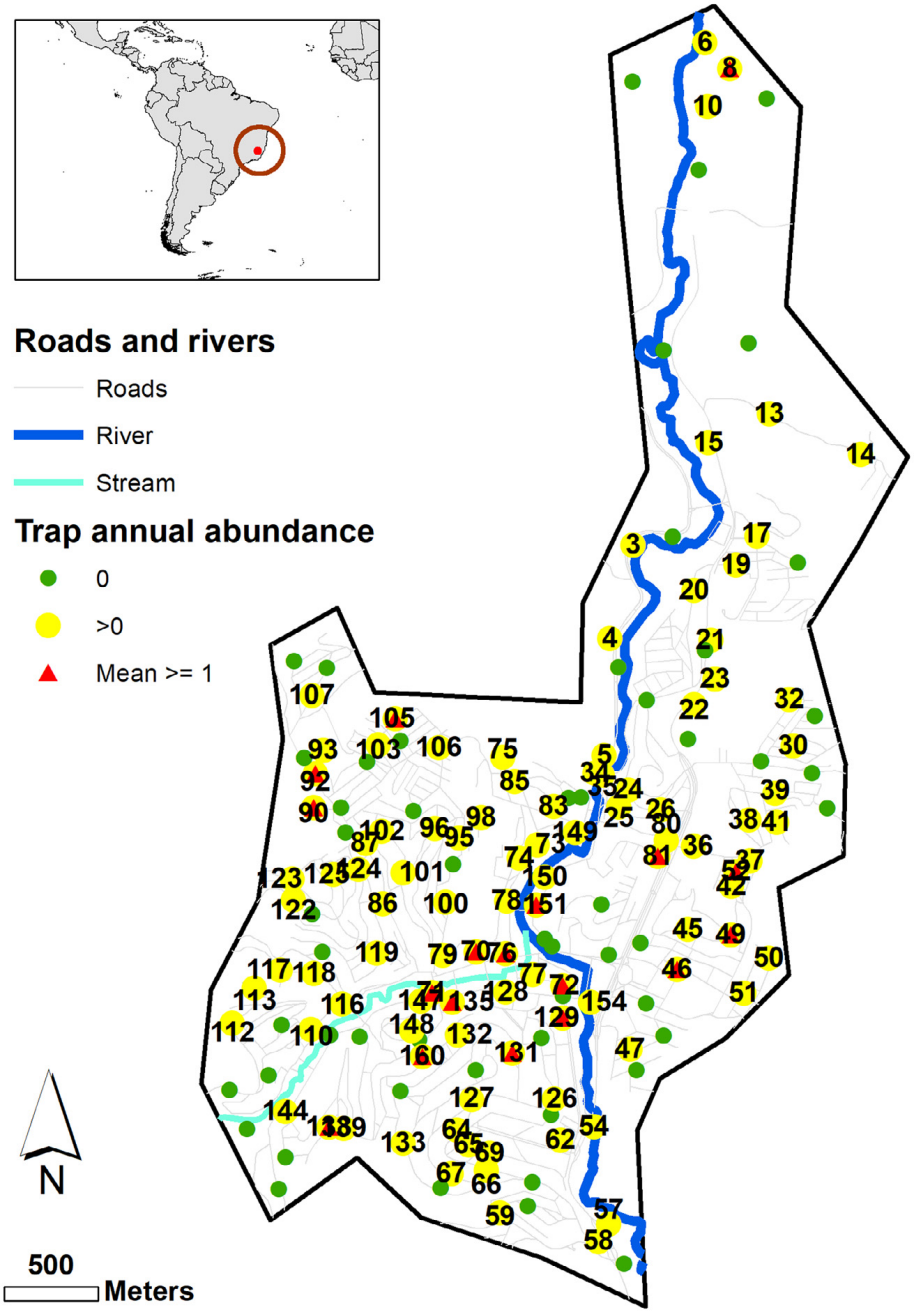
Study area in the municipality of Caratinga, Minas Gerais, Brazil. Location of the mosquito traps without (green) and with (yellow) catches of Ae. aegypti mosquito during the sampling period (2010–2011). Red triangles show those traps with average annual catches of 1 or more mosquitoes. Traps highlighted with identification number. Numeric monthly catches at each trap are shown in Table A1 in Appendix A. Roads, streams and rivers obtained from OpenStreetMap https://www.openstreetmap.org under CC BY-SA licence. World countries map from Natural Earth, CC BY-SA @ naturalearthdata.com. (For interpretation of the references to colour in this figure legend, the reader is referred to the web version of this article.)

The study was conducted in the municipality of Caratinga located in the eastern region of Minas Gerais state, Southeastern Brazil (19° 47′ 24″ S, 42° 08′ 20″ W) (Fig. 1). The study area had a total territorial area of 12 km^2^ with approximately 90,000 inhabitants. Atlantic forest is the biome of the municipality with 21°C median annual temperature and 80% humidity (data from the Brazilian Ministry of Agriculture - INMET) (Ministério da Agricultura 2015). The city is classified as a dengue endemic area, it had 1274, 91, 409 and 51 confirmed dengue cases from 2007 to 2010 respectively (data from the Brazilian Ministry of Health - SINAN) (Saúde 2015) showing a large fluctuation in dengue cases despite dengue awareness campaigns and control of dengue breeding sites (for example in 2012 there have been 55 dengue cases while in 2013 721 cases were recorded). These fluctuations are typical for infectious diseases such as dengue, where the spatial variability of the four dengue virus serotypes and the susceptibility of human population to primary and secondary infections changes over time (for Brazil see (Rodriguez-Barraquer et al., 2011; Cortes et al., 2018)).

### 2.2 Sampling design and mosquito collection data

The data was obtained using the Intelligent Aedes Monitoring System (MI-Aedes) (Ecovec, Belo Horizonte, Brazil) adopted by the Caratinga municipality (Eiras 2009), which deployed mosquito sticky traps known as MosquiTRAP (Favaro et al., 2006; Pepin et al., 2013) baited with synthetic oviposition attractant to capture gravid *Aedes* mosquitoes. 158 traps were placed at a maximum of 300 m apart following a 300 m by 300 m grid design as described in a previous work (Sedda et al., 2018). However, in practice the 300 m design was rarely achieved due to the lack of permission to locate the traps in the back or front yard of private properties and distance between traps ranged from 40 to 300 m in an irregular grid (Fig. 1).

Collection of the mosquitoes started in August 2010 and was completed in July 2011. Traps were inspected once a week and each captured mosquito was identified by species and sex (Consoli and Oliveira 1994).

### 2.3 Environmental correlates

The environmental variables used in the proposed framework were the air temperature (AT), relative humidity (RH), wet bulb temperature (WT) and atmospheric pressure (AP) as measured every 6 h by the Brazilian Instituto National de Meteorologia (http://www.inmet.gov.br) meteorological station located in the North border of Caratinga at 600 m of elevation. AT, RH and WT were the variables most significantly associated with mosquito mortality, while AT, RH, WT and AP with mosquito abundance (see Discussion). Other variables such as haziness, wind speed and wind direction, were not significant. Due to the small dimension of the area (6 × 3 km) and distances between traps, we considered only the values from the above meteorological station. However, averages for the meteorological variables at each trap differed since averages are based on the day of trap inspection (see Section 2.5).

### 2.4 Dengue cases

During the mosquito sampling campaign, 44 dengue cases were confirmed with onset of symptoms between November 2010 and July 2011. DENV1 and DENV3 were the most common dengue serotypes, while DENV2 was confirmed in only one patient. Month of symptom onset and geographic coordinates were used for this analysis, i.e. IGR and mosquito abundance were extracted at the month of onset of symptoms. Home addresses reported to health authorities were anonymized. Unlinked anonymous testing of human blood samples (where the term unlinked refer to blood samples obtained from tests not exclusively for dengue) was approved by the ethics committee in research (COEP) of Universidade Federal de Minas Gerais (number 415/04). Use of this secondary data was also approved by the Faculty of Health and Medicine Research Ethics Committee at Lancaster University (FHMREC18067).

### 2.5 Development of statistical methods

The statistical analysis was performed on the monthly sum of catches of female adult *Ae. aegypti* for each trap. AT, RH, WB and AP were monthly averaged for a month before the day of trap inspection (i.e. for the 14th October inspection, AT, RH, WB and AP were individually averaged from 14th September to 13th of October). Other monthly temporal lags (i.e. 2 and 3 months before trap inspection) were tested but their coefficients were not statistically significant (see Table A2 in Appendix A). Analyses were carried out in R-cran software (Chambers 2008).

**Fig. 2:**
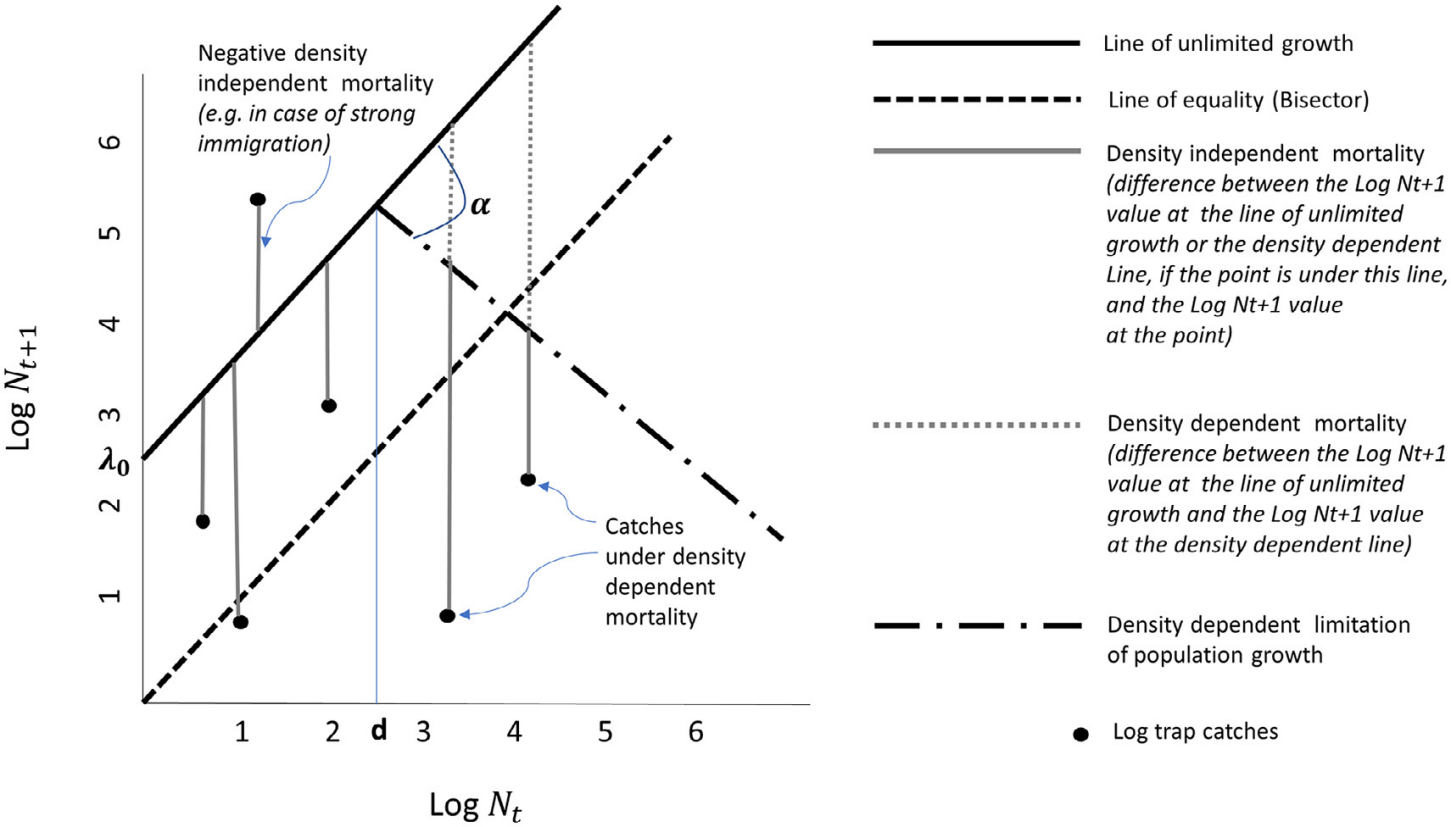
Graphical explanation of the Moran curve parameters. The density independent mortality at a mosquito log density point (log trap catches) is the difference of the log density values between the line of unlimited growth (which intercept is the unlimited growth λ_0_) and the log density point; while the density dependent mortality is the difference between the log density values between the line of unlimited growth and the line of density dependent limitation of population growth. When the density dependence is acting (densities > *d* otherwise density dependence is 0) then the density independent mortality is the difference of log density values between the line of density dependent limitation of population growth and the point. The intensity of density dependent mortality is represented by the slope α.

Population dynamics parameters are obtained from a statistical Moran curve approach, which accounts for unlimited growth rate (*λ*_0_) which modulates the density independent mortality (*DI*) and the density dependent mortality (DD). The latter can be parameterised by its intensity (*α*) and population density at which density dependent mortality starts (*d*) (Fig. 2). All these components are estimated in order to provide a measure of point IGR as described in the following sections.

#### 2.5.1 Step 1. Unlimited growth calculation

The Ricker model (Turchin 2003) describes the species abundance or density at time *t* + 1:

(1) $$N_{t+1}=\lambda_{0}N_{t}exp\left(\alpha N_{t}\right)$$

where *t* is the time step, *α* is the parameter controlling the density dependence, and *λ*_0_ is the unlimited growth. *α* implies regulation when its value is below 0 while for ln*(λ*_0_*)*<2, the population is considered stable (Hoshi et al., 2017). In statistical terms, density dependence is equivalent to the partial autocorrelation between two consecutive (in time) mosquito densities (Jian et al., 2014a). Eq. (1) can be fitted using a negative binomial distribution with over-dispersion parameter (Royle 2004), *k*:

(2) $$N_{t+1}∼NegBin\left(mean=\lambda_{0}N_{t}exp\left(\alpha N_{t}\right),overdispersion=k\right)$$

This model implies stochasticity in individuals independent of their density. In order to estimate the maximum unlimited growth, λ_0_, as defined by the Moran curve approach shown in Fig. 2, we omitted the density dependent term from Eq. (2) and estimated λ_0_ from:

(3) $$N_{t+1}∼NegBin\left(mean=\lambda_{0}N_{t},overdispersion=k\right)$$

via iteratively reweighted least squares (Hastie 2017).

### 2.5.2 Step 2. Moran curve density independent mortality

The $\hat{\lambda_{0}}$ estimated from Eq. (3), can be plugged in Eq. (4) to estimate the density independent mortality (*DI*) at each trap, *s*, and time, *t*:

(4) $$\;\text{log}\left(DI_{t,s}\right)=\hat{\lambda_{0}}+\log\left(N_{t,s}\right)-\;\log\left(N_{t+1,s}\right)$$

### 2.5.3 Step 3. Moran curve density dependent mortality

Let's first define *DT* (assimilable to the potential total mortality or k-value (Varley and Gradwell 1960)) as:

(5) $$DT_{t,s}=log\left(N_{t,s}+\hat{\lambda_{0}}\right)$$

Once this quantity has been estimated from Eq. (5) (using $\hat{\lambda_{0}}\;$from Eq. (3) and *N_t,s_* from the data), the population size at which the density dependent mortality starts, *d*, and the intensity of density dependence, *α*, can be estimated by assuming *DT* normally distributed and modelled via generalized linear mixed model (using an iteratively reweighted least squares fitting method):

(6) $$\log\left(DT_{t,s}\right)∼N\left(d+\beta X+\alpha \log\left(N_{t,s}\right)+\varphi R,\sigma^{2}\right)$$

where $\sigma^{2}$ is the constant scale parameter; **X** is a matrix of scaled (by removing the mean and dividing by the standard deviation) environmental covariates (AT, RH and WT) with regression coefficients *β*; **R** is the random effect (trap ID); and *φ* the coefficient for the random effect*.α* is commonly regarded as the intensity of the density dependent mortality. Expressing *α* in degrees clockwise from the unlimited growth line (Fig. 2):

(7) $$\alpha \;\left(degrees\right)=90-\left(\tan^{-1}\left(\alpha \right)\right)\left(\frac{180}{\pi }\right)$$

and following the interpretation of the slope from (Varley et al., 1973), a population can be classified as:

a) undercompensating, if *α* is lower than 45° (0<*b*<1 in (Varley et al., 1973))
b) exact compensating, if *α* is equal to 45° (*b* = 1 in (Varley et al., 1973))
c) overcompensating, if *α* is larger than 45° (*b*>1 in (Varley et al., 1973))

The adjustment of 90° is due to the rotation of the axis during the modeling of *d* and *α* (see (Varley et al., 1973) pages 22 and 23).

Monthly density dependence mortality values at each location are produced by the following mathematical system of equations (symbols as in Fig. 2):

(8) $$\begin{array}{c} if\;\text{log}\left(N_{t,s}\right)\leq d\\ \log\left({\hat{DD}}_{t,s}\right)=0 \end{array}$$

*else*

(9) $$\log\left({\hat{DD}}_{t,s}\right)=\left(\log\left(N_{t,s}\right)-d\right)\left(1+\tan\left(\alpha \right)\right)$$

It is therefore assumed that density dependence is a switch process, i.e. absent when densities are lower than *d*. We recognize that this assumption is inaccurate since density dependence is always present, but at negligible intensities for small population sizes (lower than *d*) (Sedda et al., 2014).

#### 2.5.4 Step 4. Obtaining intrinsic growth rate of mosquito population

In the last two steps we estimated the contribution of both density dependent and independent mortalities to changes in total generation mortality over several generations. It is important to specify that mortality (density dependent and independent) is used broadly in this context, and contains immigration and emigration processes. A strong immigration will therefore be characterized by a negative mortality (Fig. 2). At equilibrium or stability point, the difference between unlimited growth and density independent mortality is thus accounted for by density dependent mortalities (e.g. competitors, predators or parasites). If inescapable density independent losses exceed unlimited growth rate, the population will be in continuous decline until it becomes extinct. Such populations can persist in an area only if they are periodically ‘topped up’ from elsewhere by immigration. In this approach, the unlimited growth is usually assumed to be constant, this means that variations in unlimited growth are treated as variations in mortality. This is unlikely to be a serious problem because natural variations in unlimited growth (and therefore fertility) are very much less than natural variations in mortality (Sedda et al., 2014).

The adult population change in size between two consecutive time steps due to losses (mortality, emigration) and gains (fertility, immigration) (Herrando-Pérez et al., 2012) is called the intrinsic growth rate, IGR::

(10) $$IGR=\hat{\lambda_{0}}-\;\text{log}\left(DI_{t,s}\right)-\;\text{log}\left(DD_{t,s}\right)$$

with $\hat{\lambda_{0}}$ estimated from Eq. (3), *DI* estimated from Eq. (4) and *DD* estimated from Eq. (8) and Eq. (9).

#### 2.5.5 Step 5. Mosquito intrinsic growth rate and abundance mapping

Monthly mosquito intrinsic growth rate and abundance and their standard deviations at each point, *s*, and month, *t*, in a grid of 5 by 5 m were estimated.

Mosquito abundance was modelled with a zero inflated Poisson regression (ZIP) spatio-temporal kriging. In practice the residuals from a zero inflated Poisson regression on mosquito abundance were mapped via spatio-temporal kriging. Abundance at each point in the grid was obtained by summing the predicted residuals (from the kriging) with the deterministic trend surface (from the ZIP). Following the notation in (Lambert 1992) and assuming the same model of Eq. (1), the mosquito abundances *N* are independent with:

(11) $$\begin{array}{c} N_{t,s}∼0\;with\;probability\;p_{t,s}\\ N_{t,s}∼\;Poisson\left(u_{t,s}\right)\;with\;probability\;1-p_{t,s}\\ therefore\;when:\\ N_{t,s}=0\;the\;probability\;is\;p_{t,s}+\left(1-p_{t,s}\right)e^{-u_{t,s}}\\ N_{t,s}=q\;the\;probability\;is\;\left(1-p_{t,s}\right)e^{-u_{t,s}}\frac{u_{t,s}^{q}}{q!} \end{array}$$

with *q* = 1,2,…, Q. The parameters for the log linear model are defined as:

(12) $$\begin{array}{c} log\left(u\right)=\beta_{u}W\\ log\left(p\right)=\beta_{p}B \end{array}$$

with **W** and **B** covariates for the Poisson mean (**u**) (which is the count model) and the probability of the perfect state (**p**) (which is the zero-inflation model) respectively. In this work we have chosen **W** as matrix of four covariates, three common to the Moran curve analysis (air temperature, relative humidity and wet bulb temperature) and one, atmospheric pressure added since improving the model fit. Matrix **B** is a column of traps ID. Using the same covariates in **W** for **B** or other covariates do not improve the model fit. In this mixture model, the counts are modelled with a Poisson distribution, while the zero-inflation used a binomial model. The analysis was run using the function zeroinfl in pscl R package (Zeileis et al., 2008).

Residuals from the ZIP model were mapped as spatial process *Z*(*t,s*) with an ordinary spatio-temporal kriging:

(13) Z(t,s)=μ+e(t,s)

where *μ* is the mean (assumed constant over the area), E[e(t,s)]=0 and covariance, Cov(e(t,s))=V. When *V* is known, the best linear unbiased prediction of an unsampled location *Z*(*t_0_,s_0_*) is

(14) $$\begin{array}{c} \hat{Z\left(t_{0,}s_{0}\right)}=\mu +v_{0}^{\prime }V^{-1}\left(z\left(t,s\right)-\mu \right)\\ \;\;\;\;\;\;\;\;\;\;\;\;\;\;\;\;\;\;\;\;\;\;\;\;\;\;\;\;\;\;\;\;\;\;\;\;\;\;\;\;\;\;\;\;\;\;\;\;\;\;\;\;\;\;\;\;\;\;\;\;\;\;\;\;\;\;\;\text{and}\\ v_{0}=\left(Cov\left(e\left(s_{1,}t_{1}\right),e\left(s_{0,}t_{0}\right)\right),\ldots ,Cov\left(e\left(s_{n,}t_{m}\right),e\left(s_{0,}t_{0}\right)\right)\right) \end{array}$$

where all the coupled locations are within a spatial distance *ω* and temporal distance *ρ*; the covariance function (of a second order stationary process) is a separable double exponential model:

(15) $$Cov\left(\omega ,\rho \right)=\gamma^{2}exp\left\{-\frac{\Delta_{s}}{\omega }-\frac{\Delta_{t}}{\rho }\right\}$$

with spatial range *ω*, temporal range *ρ,* and the Euclidean distance Δ in space, *s*, and absolute distance Δ in time, *t*, as indicated by the subscript.

In practice, *ω* and *ρ* delimit the maximum spatial and temporal distances at which autocorrelation (and therefore smoothness) exist between two locations.

The shape of the covariance *Cov*(*ω, ρ*), and the values of, *ω*, and *ρ* were selected based on those minimizing the mean squared error. Uncertainty around these values is provided by producing the covariance envelopes. These are calculated from a spatial permutation of the data values at the trap spatial locations and by re-fitting the experimental covariance (Eq. (15)). The number of permutations was 999 (Walker et al., 1997).

The intrinsic growth rate from step 4 was mapped by employing the same ordinary spatio-temporal kriging model and covariance (double exponential) described above Eqs. (13)-(15).

Spatio-temporal ordinary kriging was performed using the function krigeST from gstat package.

#### 2.5.6 Testing the alternative

We compared our density dependence estimates with the negative binomial generalized linear model proposed by Chaves and colleagues (Chaves et al., 2015) to fit the environmental stochastic Ricker model:

(16) $$N_{t+1}∼NegBin\left(mean=\lambda_{0}N_{t}exp\left(\alpha N_{t}+\beta X_{t}\right),overdispersion=k\right)$$

This equation is one of the common approaches in modeling longitudinal data for species abundance. It allows for the estimation of unlimited growth and density dependence intensity in the presence of environmental factors. In order to account for random effects originated by repeated collections of mosquitoes from the same trap, we employed a negative binomial generalized mixed model (function glmer.nb in lme4 R package), with trap ID as random effect. In **X** we use the same covariates of Eq. (6) (AT, RH and WB) with one month lag.

#### 2.5.7 Comparing predictors for dengue cases model

We evaluated if any spatio-temporal association exists between the incidence of dengue cases and the mosquito intrinsic growth rate and abundance. To model dengue cases at the pixel level across our study region we assumed that the locations of the cases follow a spatiotemporal log-Gaussian Cox process (Taylor et al., 2018; Taylor 2019). This model assumes the number of cases, Y(*s; t*) follows a Poisson distribution:

(17) $$\begin{array}{c} Y\left(s,t\right)=Poisson\left[M\left(s,t\right)\right]\\ logM\left(s,t\right)=\log\left(P\left(s,t\right)\right)+\beta X\left(s,t\right)+G\left(s,t\right) \end{array}$$

where *P*(*s; t*) is a known component of the intensity function (the 2010 number of people per grid-cell from WORLDPOP project (Tatem 2017), which is provided at a resolution of 3 ′onds, meaning approximately 100 m), **X**(*s; t*) is a vector of covariates (mosquito abundance and/or IGR), *β* is a vector of parameter effects to be estimated and **G** is a spatiotemporal Gaussian process where covariance is modelled as in Eq. (15).

We used zero mean independent Gaussian priors for *β* with standard deviation 10^4^. For log *γ*, log *ω* and log *ρ* (as described in Eq. (15)), we used independent Gaussian priors with respective means 0, log 100 and 0 and respective standard deviations 0.3, 0.3 and 1. Our prior for *ω* gives a range of up to around 400 m, a value based on IGR and mosquito abundance modelling (Tables 1 and 2). The parameter *γ* controls the variability in the latent spatial process, **G**. We ran the MCMC algorithm for 3000,000 iterations, using a burn-in of 100,000 iterations and retaining every 2,900th sample to give us a final sample of size 1000. We checked for convergence and good mixing of our chain by examining the trace plot for each parameter and we also used a plot of the log posterior density over the iterations as a global measure of convergence (Figure B1 in Appendix B). Computation for the main analysis in this paper took place on an NVIDIA Titan XP GPU with 3840 CUDA cores and 12GB GDDR5X RAM.

**Table 1:** Intrinsic growth rate modelling. Spatio-temporal Kriging and Moran curve parameters. Uncertainties provided as envelopes for the spatio-temporal covariance parameters, and as standard errors for the Moran curve parameters. *Standardised so that the sum of the nugget and partial sill is equal to 1.

Parameters	Value	
*Kriging model: Spatio-temporal covariance*		Envelopes
*ω*, spatial range (m)	366	73,211
*ρ*, temporal range (months)	6.000	1,9
Spatial nugget*	0.169	0,0.51
Spatial partial sill*	0.831	0.5,1
Temporal nugget*	0.269	0,0.62
Temporal partial sill*	0.731	0.3,1
*Moran curve model. Coefficients from*Eq. (6)		Standard errors
Air temperature (scaled)	0.521	0.227
Wet bulb temperature (scaled)	−0.625	0.226
Relative humidity (scaled)	0.243	0.098
α, density dependence slope (degrees)	56.29	2.37
*d*, density dependence start	0.58	0.055
*Moran curve model. Parameter from*Eq. (3)*.*		Standard errors
λ_0_, field fertility (unlimited growth)	1.04	1.02

**Table 2:** Mosquito abundance modelling. Spatio-temporal Kriging and Moran curve parameters for the residuals obtained from the ZIP model described in Eq. (11) and (12). *Standardised so that the sum of the nugget and partial sill is equal to 1.

Parameters	Value	
*Kriging model: Spatio-temporal covariance*		Envelopes
*ω*, spatial range (m)	64.000	13,150
*ρ*, temporal range (months)	11.000	7,12
Spatial nugget*	0.000	0,0.1
Spatial partial sill*	1.000	0.9,1
Temporal nugget*	0.314	0,0.45
Temporal partial sill*	0.686	0.55,1
*ZIP, count part*		Standard errors
Intercept	−173.521	5.048
Air temperature (scaled)	1.213	0.424
Relative humidity (scaled)	0.173	0.062
Wet bulb temperature (scaled)	−0.907	0.458
Atmospheric pressure (scaled)	0.158	0.061

Intrinsic growth rate and dengue cases, quantifying the geographic association.

The four models evaluated here are with a null model with no **X** component (model1); ***X*** = mosquito abundance (model 2); ***X*** = (model 3); and ***X***=IGR and mosquito abundance (model 4). Each model has an intercept term and a spatio-termporal Gaussian process (random effect).

To evaluate the predictive accuracy of these Bayesian hierarchical models we have used two information criteria: Deviance Information Criterion (DIC) and Watanabe-Akaike Information Criterion (WAIC). Both measures are designed for Bayesian analyses, however WAIC averages over the posterior distribution rather than conditioning on a point estimate as in DIC (Gelman et al., 2014). This means that WAIC takes into account the predictions used for new data, while DIC takes into account the performance of the predictive density for predictions (Gelman et al., 2014). As pointed out by Vehtari et al. (2016) “*DIC can produce negative estimates of the effective number of parameters in a model and it is not defined for singular models. The WAIC is fully Bayesian in that it uses the entire posterior distribution, and it is asymptotically equal to Bayesian cross-validation. Unlike DIC, WAIC is invariant to parametrisation and also works for singular models*”. However, WAIC often produces values with small differences between models with similar structure (see for example (Gelman et al., 2014) and for an ecological application (Elderd 2017)), therefore we have decided to report both WAIC and the DIC estimates in order to provide evidence of consensus between the two statistics and report a measure, the DIC, which is familiar among Bayesian ecologists.

## 3 Results

The majority of captured *Aedes* mosquitoes were female (95.9%) and belonging to *Ae. aegypti* (88%). The *Ae. aegypti* mosquito surveillance data was characterised by a large amount of zero catches (83% of the entire dataset). The non-zero range spans from 1 to 16 *Ae. aegypti* per trap. The data is characterised by a strong seasonality with largest mosquito catches in the wet season (December to May with up to 110 *Ae. aegypti* total area catches) and lowest mosquito catches in the dry season (June to November, with maximum of 25 *Ae. aegypti* total area catches). See Table A1 in Appendix A for catches in individual traps. Finally 66% of the traps caught at least one *Ae. aegypti* mosquito during the surveillance campaign (traps indicated in yellow in Fig. 1).

### 3.1 Moran curve estimated parameters

**Fig. 3:**
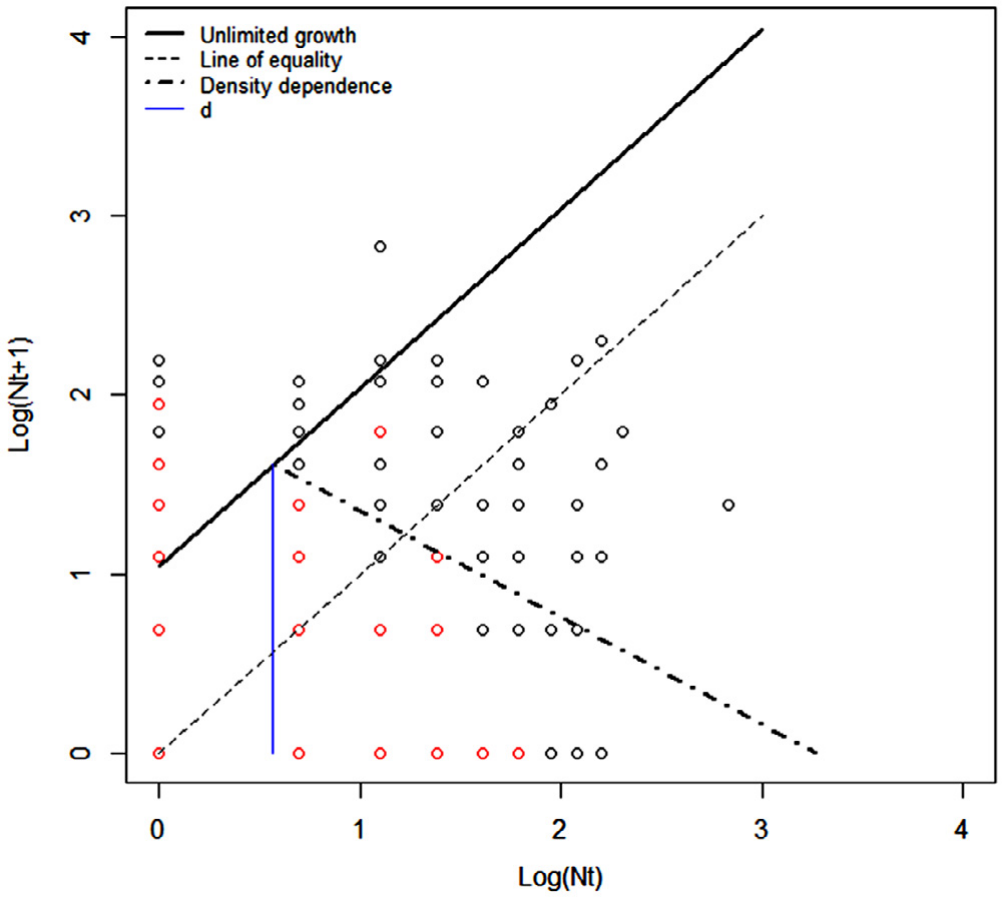
Plot of the estimated Moran Curve for *Ae. aegypti* caught in 158 traps in Caratinga. Points represent the log abundances (N) at time t (x-axis) and *t* + 1 (y-axis). Red points are highly frequent abundance combinations (i.e. occurring more than 3 times). See Figure 2 for explanation of the lines.

*Ae. aegypti* population dynamic in terms of Moran curve parameters are presented in Table 1 and visualized in Fig. 3. As per the original formalisation of the Moran Curve (Rogers 1979), Fig. 3 shows lines fitting the points and the mortalities. The lines were drawn based on the results from different models (Step 1 and 2). In Fig. 3 the unlimited growth rate line has intercept *λ_0_* (obtained from Eq. (3) (Royle 2004)) and slope of 45° (i.e. population assumed with constant unlimited growth). This line does not overlay all the points (representing mosquito abundances). Points above this line may indicate the presence of mosquito immigration (Sedda et al., 2014), however given the heterogeneity in the number of mosquitoes captured per trap, large abundances may be due to natural variation within population and unable to be explained by a 12 months survey (see Discussions). The density dependence starts at log abundance (*d*) of 0.58 with slope (α) of 56.3° (both parameters obtained from Eq. (6) assuming normal distribution for the mortality). Again, the density dependent line did not fit the points because it is modelled to fit the quantity DT (visually this would mean to rotate the unlimited growth rate as the y axis of a new plot). The slope is larger than 45° indicating an overcompensating population (see Figure 2.9 in (Varley et al., 1973)). However, not all the traps show similar values. In fact some of the traps (Table A3 in Appendix A) were under extreme overcompensation (slope larger than 90°) while others undercompensated (slope lower than 45°). Individual unlimited growth lines (Table A3 in Appendix A) were generally below the one of the general model (69% of the traps) since a large proportion of traps are affected by a higher concentration of low mosquito abundances (the combination of zero catches at time t and *t* + 1 are 661, e.g. 57% of the data points shown in Fig. 3). Similarly, the density dependence line of the general model, which is affected by large abundances, is often above the individual trap density dependent line (61% of the traps). The average slope for individual traps (60°) is close to the one of the general model (56°).

Table 1 shows the estimated coefficients, envelopes and standard errors for the parameters of *Ae. aegypti* intrinsic growth rate framework. The estimated parameters for the exponential spatio-temporal covariance are a spatial range of 336 m, and a temporal range of 6 months (Table 1), equivalent to the length of the seasons. The estimated nugget to sill ratio (a measure of explained spatial variance) is above 70% in both the spatial and temporal covariances demonstrating the presence of a strong spatial dependence (fitted by the double exponential function) and showing, once again, the importance of using spatially explicit functions in modeling mosquito population dynamics (Hassell et al., 1991; Allen et al., 2001; Cianci et al., 2015). The relatively short spatial range of the intrinsic growth rate (lower than 1 km when considering the envelopes) may indicate spatial fragmentation of mosquito-related local factors (breeding sites, humans and their settlements etc.). The covariates are statistically significant and coefficient signs are coherent with the biology of *Ae. aegypti* (see Discussion) (Derrick and Bicks 1958; Li et al., 2019).

### 3.2 Alternative model

The significant presence of density dependence was also confirmed using a negative binomial generalized mixed linear model as adapted from Chaves and colleagues (Chaves et al., 2015) to fit the environmental stochastic Ricker model (Eq. (16)). We obtained a density dependence slope equivalent to 78.35° (*p*-value <0.001) confirming an overcompensating population, although with an intensity stronger than the one obtained by the Moran Curve approach. The covariates coefficients were identical to the mortalities in the intrinsic growth rate framework, however not statistically significant (*p*-value > 0.05). Summary statistics of the model are shown in Table A4 in Appendix A.

### 3.3 Mosquito intrinsic growth rate and abundance

The intrinsic growth rate is spatially heterogeneous with minimum value of 0, and maximum of 3.66 (a 38 fold increase, as obtained by exponentiating 3.66). The average intrinsic growth rate for the 12 months period was 0.34, a value showing a slight increase in the population (zero is equivalent to birth rate = death rate)(Trajer et al., 2017; Chaves and Moji 2018).

Mosquito abundance ZIP regression spatio-temporal kriging model parameters are summarised in Table 2. Mosquito abundance is characterised by a shorter spatial range and longer temporal range than mosquito intrinsic growth rate, which describes a larger spatial heterogeneity and temporal homogeneity than the intrinsic growth rate. The covariate effect's is similar to the one found in the intrinsic growth rate framework (compare coefficients in Tables 1 and 2).

The compared models: (i) With no predictors; (ii) mosquito abundance as predictor; (iii) intrinsic growth rate as predictor; (iv) intrinsic growth rate and density of mosquitoes as predictors; all containing a spatio-temporal Gaussian process and an intercept term, were compared using two information criteria, the WAIC and DIC(Gelman et al., 2014). Table 3 shows the summary statistics for the four models.

**Table 3:** Summary statistics for the spatio-temporal log-Cox Gaussian process used to model the dengue cases. In parenthesis the 95% credible interval are reported for each parameter.

Parameter	Model 1: Intercept only	Model 2: Intercept and Abundance	Model 3: Intercept and IGR	Model 4: Intercept, Abundance and IGR
*β*_intercept_	1.6 10^−8^ (1 10^−8^, 2.2 10^−8^)	1.8 10^−8^ (9 10^−9^, 3.1 10^−8^)	1.6 10^−8^ (1 10^−8^, 2.6 10^−8^)	1.8 10^−8^ (9 10^−9^, 3 10^−8^)
*β*_Abundance_	–	0.84 (0.18,1.9)	–	0.99 (0.09,3.9)
*β*_IGR_	–	–	0.91 (0.33,1.8)	1.30 (0.25,3.9)
γ	1.9 (1.3,2.5)	1.9 (1.4,2.5)	1.9 (1.4,2.5)	1.9 (1.4,2.6)
Ω	138 (78,215)	136 (82,210)	135 (78,209)	136 (79,214)
*Ρ*	8.1 (4.5,20)	7.9 (4.5,19)	7.8 (4.5,18)	7.6 (4.5,16)
WAIC	504.43	505.34	503.88	508.12
DIC	1018.33	1013.58	976.10	958.24

**Fig. 4:**
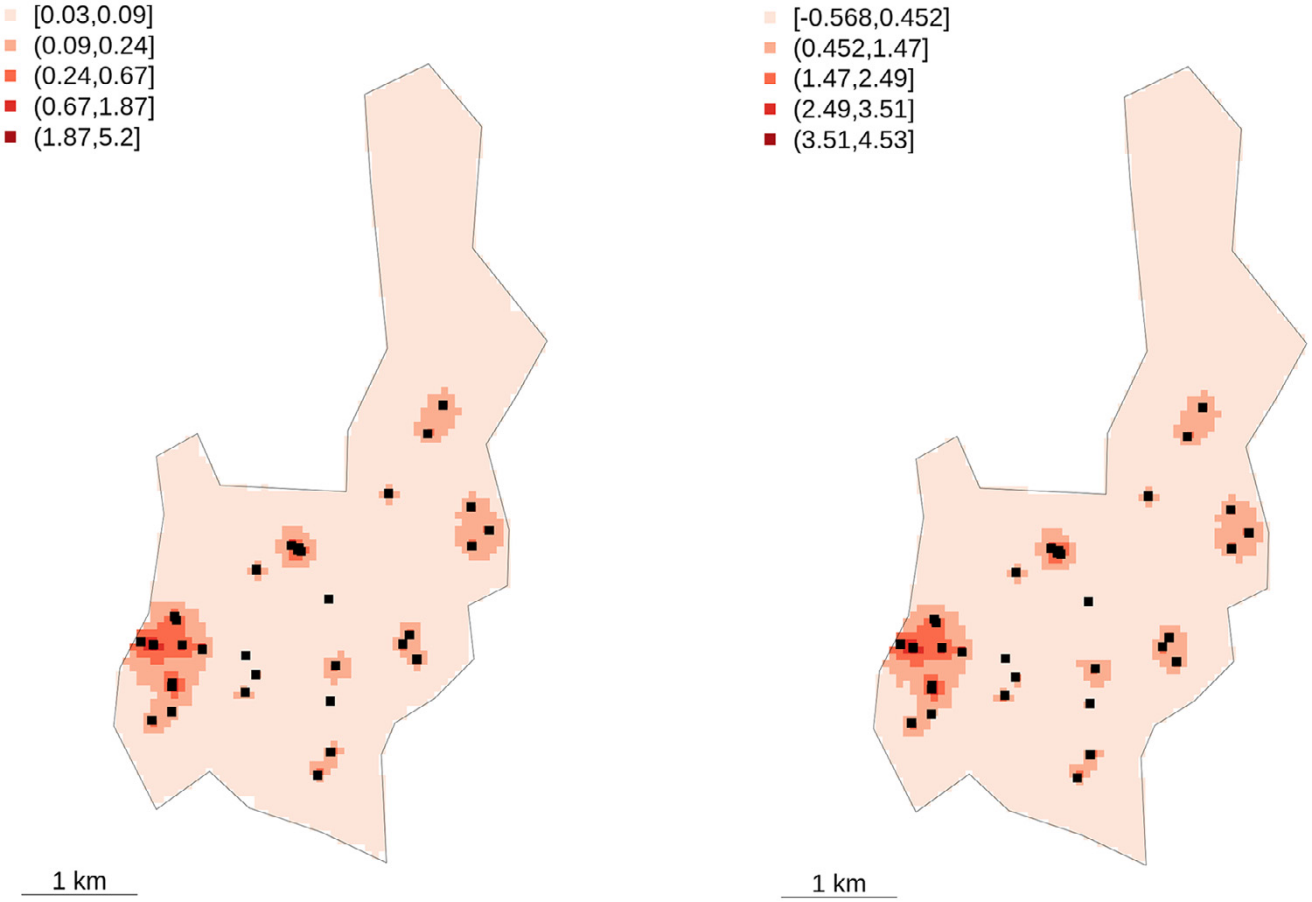
Weighted average of predicted dengue cases x 1000 people during the 2011/12 surveillance campaign (left). On the right is shown the equivalent exceedance map, with exceedance threshold of 0.33 × 1000 people. (For interpretation of the references to colour in this figure legend, the reader is referred to the web version of this article.)

A smaller WAIC and DIC indicates better model predictive accuracy. In both WAIC and DIC model 3 (IGR) outperforms model 2 (abundance). Smaller differences are found between model 1 (intercept only) and model 2. The two information criteria disagree with model 4, ranked as the worst by WAIC and as the best by DIC. However, due to the structure of the model and the approximation to cross-validation of the WAIC penalty term, the WAIC must be considered a more reliable measure compared to DIC. Based on WAIC the order of preference is for models 3, which contains the intercept, random effect and IGR, followed by model 1 (containing the intercept and random effect). Model 2 (intercept, random effect and mosquito abundance), and model 3 (intercept, random effect, mosquito abundance and IGR), do not outperform the null model (model 1) based on WAIC statistic. The averaged predicted number of dengue cases and exceedances for the Caratinga area are given in Fig. 4. The risk is geographically limited around the cases.

## 4 Discussion

This study shows that the geographic and temporal association of dengue incidence and mosquito intrinsic growth rate is more robust than with mosquito abundance. The latter was found to have a lower predictive accuracy than the former (in terms of WAIC and DIC) and with small differences compared to the null model. The WAIC and DIC are discordant for the model with both variables (abundance + growth) which may indicate a better inference in model 4 than model 3 but with a lower predictive accuracy (Gelman et al., 2014). It also shows that dengue incidence presents a strong spatio-temporal dependence as inferred by the statistically significant parameters employed in the spatio-temporal Gaussian process of the log-Cox model.

It is important to note that this analysis was based on symptomatic dengue cases, i.e. people tested after onset of symptoms, and therefore ignores the characteristics and patterns of dengue infections (ascertained from a serosurveillance campaign by Li and colleagues (Li et al., 2019)). In fact, a great proportion of infections are likely to be asymptomatic (Slavov et al., 2019) and the incidence of dengue cases doesn't necessarily correlate with the underlying transmission intensity (Perkins et al., 2014). In addition, we cannot unrealistically assume that all the infections originated at household location/proximity without considering other important variables that may have played a role in the geographic ranges of dengue transmission, such as house quality and socioeconomic conditions (Farinelli et al., 2018; Lippi et al., 2018) or human behaviour and mobility (shopping, schooling for example)(Stoddard et al., 2013; Chuang et al., 2018; Kraemer et al., 2018; Sanna et al., 2018; Wen et al., 2018). Road network density may also be an important factor (Li et al., 2018) and additional analyses are necessary to confirm the spatial pattern found in this work.

Our results are not surprising in the light of conventional disease transmission indices. In fact, the positive association of incidence of dengue cases with the intrinsic growth rate has a similar interpretation as for vectorial capacity and the reproductive number R0. These measures also contain some of the parameters used to calculate intrinsic growth rate (vector mortality), but do not usually account for the additional parameters such as unlimited growth and density dependent and independent mortality. The main advantage in mapping the intrinsic growth rate with the method presented here, when mosquito surveillance data is available, is that it does not require assumptions of mortality or other population parameters (Desenclos 2011).

Our results suggest (also by using the alternative model comparison) that there may be density dependent mortality for adult *Ae. aegypti* and this has important implications for *Aedes* control (Hancock et al., 2016). However, only a year of surveillance may not have differentiated density dependence from seasonal or larger periodic environmental determinants (Vincenti-Gonzalez et al., 2018). As can be expected from trapping gravid mosquito females, the unlimited growth of 1.04 is well below the theoretical log fertility rate for mosquito (between 4 and 5, when considering 100–200 eggs every 2–3 weeks) (Clemons et al., 2010; Robert et al., 2012; Robert et al., 2014). Finally, our results have shown the presence of a mixture of trap populations of different size (Table A3 in Appendix A). Discrepancies between the general (or global) model and results from individual traps may raise concerns about local heterogeneities and therefore the validity of the general model when applied to all the trap locations. This concern may be strengthened by the evidence that local and independent populations are acting in the area, however this does not seem the case for two reasons: (i) Previous analyses on the area estimated large scale (for the size of the area) mosquito mobility (Sedda et al., 2018) which may be at the origin of a metapopulation for the area of Caratinga; (ii) the overall population has a balanced average growth rate which is at the base of the definition of population (see for example (Berryman 1999) page 14). In practice, assuming a single mosquito population or metapopulation in the area of Caratinga, means modeling the population under maximum limiting factors (see again (Berryman 1999) fifth principle of population dynamics ‘limiting factors’) which is plausible for a relatively small area (12 km^2^).

Not surprisingly temperature and humidity are significantly associated with mosquito adult's mortality. Temperature affects the growth, development and survival of mosquitoes (Attaway et al., 2017) and is recognised as the most influential predictor of *Aedes* abundance (Weetman et al., 2018), as for most ectotherms, warmer temperatures reduces the size but increases the development rate of *Ae. aegypti* in the aquatic stages (Padmanabha et al., 2012) (within certain limits (Wang et al., 2016; Whiten and Peterson 2016)). Wet bulb temperature is a measure based on both temperature and humidity. No previous studies analysed the relationship between wet bulb temperature and *Ae. aegypti* apart from using it as limiting factor (Derrick and Bicks 1958). The negative association may be related to the effect of lower temperature at lower humidity levels. For the latter, the variable relative humidity has been found to increase mortality, again a common association within certain limits (Ferreira et al., 2017; Lega et al., 2017). The importance of the atmospheric pressure in the zero-inflated model employed for mosquito abundance is related to the increase in mortality of *Ae. aegypti* at lower atmospheric pressure (Galun and Fraenkel 1961). The significance of these covariates in our frameworks confirms their importance for *Ae. aegypti* and dengue modeling (Li et al., 2019).

This analysis is based on mapping the intrinsic growth rate observed for female *Aedes* mosquitoes. Recent research shows the adequacy and sometimes the superiority of female mosquito-based indices instead of egg and larvae-based measures to predict dengue risk (Parra et al., 2018). Certainly the spatial and temporal ranges found in this work need to be considered carefully before any generalisation (unless as prior-information for future Bayesian framework). These values are related to unexplained environmental conditions which may change over time. Despite this limitation, the intrinsic growth rate can be used for the accurate estimation of mosquito sources or reservoir areas which are a priority for the deployment of mosquito control measures and precision public health (Eiras 2009, Pepin et al., 2013, Dowell et al., 2016).

It is important to note that this work has some limitations. As stated above, we only used 12 months of data which may be not long enough to have full discrimination of density dependence mortality (Hoshi et al., 2014). Sampling protocols did not detect all individuals at a site, and detection rates may vary among sites, confounding abundance estimates. In addition, this work only considered few climatic variables as environmental forces on mortality. However, food limitation and/or resource competition are major determinants of the rate of *Ae. aegypti* production (Alto et al., 2012; Riley et al., 2015; Wen et al., 2015; Ruiz-Moreno 2016).

Finally, the dengue outbreak was somewhat limited in size (only 44 dengue cases were analysed), and therefore the results of the spatio-temporal Gaussian log-cox model must be treated with caution. In addition, we cannot exclude that other mosquito abundance-based metrics (ratio of abundance between two periods of time or abundance greater than a given threshold) may perform better than intrinsic growth rate. This work concentrates on the simple comparison between intrinsic growth rate and abundance which are the most common describer used for population dynamics and population distribution/suitability respectively.

## 5 Conclusions

Achieving the global dengue control strategy (the major and most diffuse disease spread by *Aedes*), which called for at least 50% reduction in the disease mortality burden and a minimum of 25% reduction in incidence by 2020 (World Health Organization 2012), requires innovative approaches and interventions that go beyond simple disease surveillance or ecological analyses. In this paper, we propose a biological-statistical framework that may serve for vector control spatial targeting. Our aim is to improve current vector surveillance programmes that are lacking in effective measurement of certain vector indicators, and we hope to promote a debate among biologists, mathematicians/statisticians and field entomologists about the optimal vector indices required for vector-borne disease control, elimination and eradication. Many of the mosquito-borne diseases do not have effective vaccines or treatments and are controlled by mosquito elimination (Robert et al., 2014; Evans et al., 2015), or by preventing vector contact (Reyes-Solis et al., 2014). The same framework can support successful interventions by identifying mosquito sources. For example, this could be applied to field trials with *Wolbachia* infected mosquitoes to reduce dengue transmission (Caragata et al., 2016; Achee et al., 2019). The present results require large scale investigations to confirm the significance of our study. The implications are obvious in terms of disease control, but also for disease surveillance and forecasting (Schwab et al., 2018), especially for a disease like dengue that has a high probability of spatial recurrence in urban areas (Chen et al., 2018).

## CRediT authorship contribution statement

**Luigi Sedda:** Conceptualization, Software, Formal analysis, Validation, Investigation, Visualization, Methodology, Writing - original draft, Writing - review & editing. **Benjamín M. Taylor:** Formal analysis, Validation, Methodology, Writing - original draft, Writing - review & editing. **Alvaro E. Eiras:** Conceptualization, Writing - original draft, Writing - review & editing. **João Trindade Marques:** Conceptualization, Resources, Data curation, Investigation, Writing - original draft, Project administration, Writing - review & editing. **Rod J. Dillon:** Methodology, Writing - original draft, Writing - review & editing.

## Declaration of Competing Interest

The authors declare that they have no known competing financial interests or personal relationships that could have appeared to influence the work reported in this paper.

## Appendix Supplementary materials

Image, application 1

Image, application 2
