# Supplementary material for: Using the intrinsic growth rate of the mosquito population improves spatio-temporal dengue risk estimation
Source: Acta Trop. 2020 Aug;208:105519. doi: 10.1016/j.actatropica.2020.105519 (PMC7315132; doi:10.1016/j.actatropica.2020.105519)
Supplement: Supplementary file 2 [file mmc2.docx]

## Appendix A. Supplementary Tables

Table A1. Number of caught Ae. aegypti per trap and per month and relative statistics.

| Trap ID | Jan | Feb | Mar | Apr | May | Jun | Jul | Aug | Sep | Oct | Nov | Dec | Min | Mean | Max |
| --- | --- | --- | --- | --- | --- | --- | --- | --- | --- | --- | --- | --- | --- | --- | --- |
| 3 | 0 | 1 | 0 | 0 | 0 | 0 | 0 | 0 | 0 | 0 | 0 | 0 | 0 | 0.08 | 1 |
| 4 | 0 | 0 | 0 | 2 | 0 | 0 | 0 | 0 | 0 | 0 | 0 | 0 | 0 | 0.17 | 2 |
| 5 | 0 | 0 | 2 | 0 | 0 | 0 | 0 | 0 | 1 | 1 | 1 | 0 | 0 | 0.42 | 2 |
| 6 | 0 | 0 | 2 | 0 | 0 | 0 | 0 | 0 | 0 | 0 | 0 | 0 | 0 | 0.17 | 2 |
| 8 | 0 | 6 | 6 | 0 | 2 | 0 | 0 | 0 | 1 | 1 | 0 | 6 | 0 | 1.83 | 6 |
| 10 | 0 | 0 | 0 | 0 | 0 | 0 | 0 | 0 | 1 | 0 | 0 | 0 | 0 | 0.08 | 1 |
| 13 | 0 | 0 | 0 | 0 | 0 | 1 | 0 | 0 | 0 | 0 | 0 | 0 | 0 | 0.08 | 1 |
| 14 | 0 | 0 | 0 | 1 | 0 | 0 | 0 | 0 | 0 | 0 | 0 | 0 | 0 | 0.08 | 1 |
| 15 | 0 | 1 | 0 | 1 | 0 | 0 | 0 | 0 | 0 | 0 | 0 | 0 | 0 | 0.17 | 1 |
| 17 | 0 | 0 | 0 | 0 | 0 | 0 | 0 | 1 | 0 | 0 | 0 | 0 | 0 | 0.08 | 1 |
| 19 | 0 | 1 | 1 | 5 | 1 | 0 | 0 | 0 | 0 | 0 | 0 | 1 | 0 | 0.75 | 5 |
| 20 | 0 | 0 | 0 | 1 | 0 | 0 | 0 | 1 | 0 | 1 | 0 | 0 | 0 | 0.25 | 1 |
| 21 | 0 | 0 | 0 | 0 | 1 | 0 | 0 | 0 | 0 | 0 | 0 | 0 | 0 | 0.08 | 1 |
| 22 | 0 | 0 | 0 | 0 | 1 | 0 | 0 | 0 | 0 | 0 | 0 | 0 | 0 | 0.08 | 1 |
| 23 | 0 | 2 | 1 | 0 | 0 | 0 | 0 | 0 | 0 | 0 | 0 | 1 | 0 | 0.33 | 2 |
| 24 | 1 | 0 | 3 | 0 | 1 | 2 | 0 | 1 | 0 | 0 | 0 | 1 | 0 | 0.75 | 3 |
| 25 | 0 | 3 | 0 | 1 | 0 | 0 | 0 | 1 | 0 | 0 | 0 | 1 | 0 | 0.50 | 3 |
| 26 | 0 | 0 | 0 | 1 | 0 | 0 | 0 | 0 | 0 | 0 | 0 | 0 | 0 | 0.08 | 1 |
| 30 | 1 | 3 | 0 | 1 | 0 | 0 | 0 | 0 | 0 | 0 | 0 | 4 | 0 | 0.75 | 4 |
| 32 | 1 | 0 | 0 | 2 | 0 | 0 | 0 | 1 | 0 | 0 | 0 | 0 | 0 | 0.33 | 2 |
| 34 | 0 | 2 | 0 | 0 | 0 | 0 | 0 | 0 | 0 | 0 | 1 | 0 | 0 | 0.25 | 2 |
| 35 | 0 | 0 | 0 | 0 | 1 | 0 | 0 | 1 | 0 | 0 | 0 | 1 | 0 | 0.25 | 1 |
| 36 | 0 | 0 | 2 | 1 | 0 | 1 | 0 | 1 | 0 | 0 | 0 | 1 | 0 | 0.50 | 2 |
| 37 | 0 | 0 | 0 | 0 | 0 | 0 | 0 | 0 | 0 | 0 | 0 | 1 | 0 | 0.08 | 1 |
| 38 | 2 | 0 | 0 | 0 | 0 | 0 | 0 | 0 | 0 | 0 | 0 | 0 | 0 | 0.17 | 2 |
| 39 | 5 | 0 | 0 | 1 | 0 | 0 | 0 | 1 | 0 | 1 | 0 | 0 | 0 | 0.67 | 5 |
| 41 | 0 | 2 | 0 | 0 | 0 | 0 | 0 | 1 | 0 | 0 | 0 | 0 | 0 | 0.25 | 2 |
| 42 | 2 | 0 | 0 | 0 | 0 | 0 | 0 | 0 | 0 | 0 | 0 | 1 | 0 | 0.25 | 2 |
| 45 | 0 | 0 | 0 | 0 | 0 | 1 | 0 | 0 | 0 | 0 | 0 | 0 | 0 | 0.08 | 1 |
| 46 | 1 | 0 | 1 | 7 | 1 | 0 | 0 | 0 | 0 | 0 | 0 | 4 | 0 | 1.17 | 7 |
| 47 | 1 | 0 | 0 | 0 | 0 | 0 | 0 | 0 | 0 | 0 | 0 | 0 | 0 | 0.08 | 1 |
| 49 | 3 | 2 | 3 | 0 | 1 | 0 | 0 | 0 | 1 | 1 | 1 | 3 | 0 | 1.25 | 3 |
| 50 | 1 | 0 | 0 | 1 | 0 | 0 | 0 | 0 | 0 | 0 | 0 | 0 | 0 | 0.17 | 1 |
| 51 | 0 | 0 | 0 | 0 | 0 | 0 | 0 | 1 | 0 | 0 | 0 | 0 | 0 | 0.08 | 1 |
| 52 | 2 | 1 | 0 | 0 | 0 | 1 | 0 | 1 | 0 | 0 | 0 | 7 | 0 | 1.00 | 7 |
| 54 | 4 | 2 | 0 | 0 | 1 | 0 | 0 | 0 | 0 | 1 | 1 | 1 | 0 | 0.83 | 4 |
| 57 | 0 | 0 | 0 | 1 | 1 | 0 | 0 | 0 | 0 | 0 | 0 | 2 | 0 | 0.33 | 2 |
| 58 | 0 | 0 | 0 | 0 | 0 | 0 | 0 | 0 | 0 | 0 | 0 | 1 | 0 | 0.08 | 1 |
| 59 | 0 | 0 | 0 | 0 | 0 | 2 | 0 | 1 | 0 | 0 | 0 | 0 | 0 | 0.25 | 2 |
| 62 | 0 | 0 | 0 | 0 | 1 | 0 | 0 | 0 | 0 | 0 | 0 | 0 | 0 | 0.08 | 1 |
| 64 | 1 | 1 | 0 | 1 | 0 | 0 | 0 | 0 | 1 | 0 | 1 | 1 | 0 | 0.50 | 1 |
| 65 | 5 | 0 | 0 | 0 | 0 | 0 | 0 | 4 | 0 | 1 | 0 | 1 | 0 | 0.92 | 5 |
| 66 | 0 | 3 | 1 | 1 | 0 | 0 | 0 | 0 | 0 | 0 | 0 | 1 | 0 | 0.50 | 3 |
| 67 | 0 | 0 | 1 | 0 | 0 | 1 | 0 | 0 | 0 | 0 | 0 | 0 | 0 | 0.17 | 1 |
| 69 | 0 | 0 | 0 | 0 | 0 | 0 | 0 | 0 | 0 | 0 | 0 | 1 | 0 | 0.08 | 1 |
| 70 | 0 | 3 | 1 | 1 | 1 | 2 | 0 | 1 | 0 | 1 | 0 | 3 | 0 | 1.08 | 3 |
| 71 | 2 | 7 | 0 | 0 | 0 | 0 | 0 | 0 | 0 | 2 | 0 | 1 | 0 | 1.00 | 7 |
| 72 | 2 | 5 | 5 | 1 | 1 | 0 | 0 | 0 | 0 | 0 | 0 | 0 | 0 | 1.17 | 5 |
| 73 | 0 | 0 | 0 | 1 | 1 | 0 | 0 | 0 | 0 | 0 | 0 | 0 | 0 | 0.17 | 1 |
| 74 | 0 | 0 | 0 | 0 | 0 | 1 | 1 | 0 | 0 | 0 | 0 | 2 | 0 | 0.33 | 2 |
| 75 | 0 | 0 | 1 | 0 | 0 | 0 | 0 | 0 | 0 | 0 | 0 | 0 | 0 | 0.08 | 1 |
| 76 | 3 | 1 | 1 | 6 | 1 | 0 | 3 | 0 | 0 | 0 | 0 | 2 | 0 | 1.42 | 6 |
| 77 | 1 | 1 | 1 | 0 | 0 | 0 | 0 | 0 | 0 | 0 | 1 | 0 | 0 | 0.33 | 1 |
| 78 | 0 | 0 | 0 | 5 | 0 | 0 | 0 | 1 | 0 | 0 | 0 | 0 | 0 | 0.50 | 5 |
| 79 | 0 | 0 | 1 | 1 | 0 | 0 | 0 | 0 | 0 | 0 | 0 | 2 | 0 | 0.33 | 2 |
| 80 | 0 | 0 | 1 | 0 | 0 | 0 | 0 | 0 | 0 | 0 | 0 | 0 | 0 | 0.08 | 1 |
| 81 | 3 | 2 | 0 | 2 | 5 | 0 | 0 | 1 | 0 | 0 | 0 | 0 | 0 | 1.08 | 5 |
| 83 | 1 | 5 | 3 | 1 | 1 | 0 | 0 | 0 | 0 | 0 | 0 | 0 | 0 | 0.92 | 5 |
| 85 | 0 | 4 | 0 | 0 | 0 | 0 | 0 | 0 | 0 | 0 | 0 | 3 | 0 | 0.58 | 4 |
| 86 | 0 | 0 | 0 | 0 | 0 | 0 | 0 | 0 | 4 | 0 | 0 | 0 | 0 | 0.33 | 4 |
| 87 | 0 | 1 | 1 | 1 | 0 | 0 | 0 | 0 | 0 | 0 | 0 | 0 | 0 | 0.25 | 1 |
| 90 | 0 | 4 | 1 | 3 | 0 | 1 | 0 | 1 | 0 | 0 | 0 | 8 | 0 | 1.50 | 8 |
| 92 | 0 | 3 | 0 | 6 | 1 | 0 | 1 | 0 | 1 | 0 | 0 | 4 | 0 | 1.33 | 6 |
| 93 | 0 | 0 | 0 | 0 | 1 | 0 | 0 | 0 | 0 | 0 | 0 | 0 | 0 | 0.08 | 1 |
| 95 | 0 | 0 | 0 | 1 | 0 | 0 | 0 | 0 | 0 | 0 | 0 | 0 | 0 | 0.08 | 1 |
| 96 | 0 | 1 | 0 | 0 | 0 | 0 | 1 | 0 | 0 | 0 | 0 | 1 | 0 | 0.25 | 1 |
| 98 | 0 | 0 | 0 | 0 | 0 | 0 | 2 | 0 | 0 | 0 | 0 | 0 | 0 | 0.17 | 2 |
| 100 | 0 | 1 | 0 | 0 | 0 | 0 | 0 | 0 | 0 | 0 | 0 | 0 | 0 | 0.08 | 1 |
| 101 | 0 | 1 | 0 | 0 | 0 | 0 | 0 | 0 | 0 | 0 | 0 | 0 | 0 | 0.08 | 1 |
| 102 | 0 | 0 | 1 | 0 | 0 | 0 | 0 | 0 | 0 | 0 | 0 | 1 | 0 | 0.17 | 1 |
| 103 | 0 | 0 | 1 | 1 | 0 | 1 | 1 | 0 | 1 | 0 | 0 | 0 | 0 | 0.42 | 1 |
| 105 | 1 | 2 | 2 | 5 | 1 | 0 | 0 | 1 | 0 | 0 | 0 | 0 | 0 | 1.00 | 5 |
| 106 | 3 | 1 | 0 | 1 | 0 | 0 | 0 | 0 | 0 | 0 | 0 | 2 | 0 | 0.58 | 3 |
| 107 | 0 | 0 | 0 | 1 | 0 | 0 | 1 | 0 | 1 | 0 | 0 | 1 | 0 | 0.33 | 1 |
| 110 | 0 | 1 | 0 | 0 | 0 | 0 | 0 | 0 | 0 | 0 | 0 | 0 | 0 | 0.08 | 1 |
| 112 | 0 | 0 | 0 | 0 | 0 | 1 | 0 | 0 | 0 | 1 | 0 | 0 | 0 | 0.17 | 1 |
| 113 | 0 | 0 | 1 | 0 | 0 | 0 | 0 | 0 | 0 | 0 | 0 | 0 | 0 | 0.08 | 1 |
| 116 | 1 | 0 | 0 | 1 | 0 | 0 | 0 | 0 | 0 | 0 | 0 | 1 | 0 | 0.25 | 1 |
| 117 | 0 | 0 | 0 | 0 | 0 | 0 | 0 | 0 | 1 | 0 | 0 | 0 | 0 | 0.08 | 1 |
| 118 | 0 | 0 | 0 | 0 | 0 | 0 | 0 | 0 | 0 | 1 | 0 | 0 | 0 | 0.08 | 1 |
| 119 | 0 | 1 | 0 | 0 | 0 | 1 | 0 | 0 | 0 | 0 | 0 | 0 | 0 | 0.17 | 1 |
| 122 | 0 | 2 | 0 | 1 | 0 | 0 | 0 | 0 | 0 | 0 | 0 | 0 | 0 | 0.25 | 2 |
| 123 | 0 | 0 | 1 | 1 | 0 | 0 | 0 | 0 | 1 | 0 | 0 | 2 | 0 | 0.42 | 2 |
| 124 | 0 | 2 | 0 | 3 | 0 | 0 | 1 | 0 | 0 | 0 | 0 | 0 | 0 | 0.50 | 3 |
| 125 | 0 | 0 | 0 | 0 | 0 | 0 | 0 | 0 | 0 | 0 | 1 | 0 | 0 | 0.08 | 1 |
| 126 | 0 | 3 | 1 | 0 | 0 | 0 | 0 | 0 | 0 | 0 | 0 | 0 | 0 | 0.33 | 3 |
| 127 | 0 | 0 | 0 | 0 | 0 | 0 | 0 | 0 | 1 | 0 | 0 | 0 | 0 | 0.08 | 1 |
| 128 | 0 | 1 | 1 | 0 | 1 | 0 | 0 | 0 | 0 | 0 | 0 | 0 | 0 | 0.25 | 1 |
| 129 | 1 | 3 | 8 | 9 | 5 | 4 | 3 | 0 | 1 | 1 | 2 | 1 | 0 | 3.17 | 9 |
| 131 | 1 | 2 | 0 | 3 | 0 | 0 | 0 | 0 | 0 | 2 | 0 | 6 | 0 | 1.17 | 6 |
| 132 | 0 | 0 | 0 | 3 | 0 | 0 | 0 | 0 | 0 | 0 | 0 | 1 | 0 | 0.33 | 3 |
| 133 | 0 | 0 | 0 | 1 | 0 | 0 | 0 | 1 | 0 | 0 | 0 | 0 | 0 | 0.17 | 1 |
| 135 | 3 | 7 | 8 | 2 | 8 | 4 | 0 | 1 | 3 | 1 | 2 | 16 | 0 | 4.58 | 16 |
| 138 | 5 | 2 | 1 | 4 | 0 | 0 | 0 | 0 | 0 | 0 | 3 | 3 | 0 | 1.50 | 5 |
| 139 | 0 | 2 | 0 | 0 | 0 | 0 | 2 | 0 | 0 | 0 | 0 | 0 | 0 | 0.33 | 2 |
| 140 | 0 | 0 | 0 | 0 | 0 | 0 | 2 | 0 | 0 | 0 | 0 | 0 | 0 | 0.17 | 2 |
| 144 | 0 | 2 | 0 | 0 | 0 | 0 | 0 | 0 | 0 | 0 | 0 | 0 | 0 | 0.17 | 2 |
| 147 | 0 | 0 | 0 | 1 | 0 | 0 | 1 | 0 | 3 | 1 | 0 | 0 | 0 | 0.50 | 3 |
| 148 | 0 | 3 | 2 | 0 | 0 | 0 | 1 | 0 | 1 | 0 | 0 | 0 | 0 | 0.58 | 3 |
| 149 | 0 | 0 | 0 | 0 | 0 | 0 | 0 | 0 | 1 | 0 | 0 | 3 | 0 | 0.33 | 3 |
| 150 | 0 | 3 | 2 | 0 | 5 | 0 | 0 | 0 | 0 | 0 | 0 | 0 | 0 | 0.83 | 5 |
| 151 | 0 | 2 | 2 | 0 | 2 | 0 | 1 | 2 | 0 | 0 | 2 | 2 | 0 | 1.08 | 2 |
| 154 | 0 | 0 | 0 | 0 | 0 | 0 | 0 | 0 | 0 | 0 | 1 | 0 | 0 | 0.08 | 1 |
| 160 | 3 | 2 | 4 | 7 | 3 | 1 | 0 | 0 | 1 | 1 | 2 | 5 | 0 | 2.42 | 7 |
| 1 | 0 | 0 | 0 | 0 | 0 | 0 | 0 | 0 | 0 | 0 | 0 | 0 | 0 | 0.00 | 0 |
| 2 | 0 | 0 | 0 | 0 | 0 | 0 | 0 | 0 | 0 | 0 | 0 | 0 | 0 | 0.00 | 0 |
| 7 | 0 | 0 | 0 | 0 | 0 | 0 | 0 | 0 | 0 | 0 | 0 | 0 | 0 | 0.00 | 0 |
| 9 | 0 | 0 | 0 | 0 | 0 | 0 | 0 | 0 | 0 | 0 | 0 | 0 | 0 | 0.00 | 0 |
| 11 | 0 | 0 | 0 | 0 | 0 | 0 | 0 | 0 | 0 | 0 | 0 | 0 | 0 | 0.00 | 0 |
| 12 | 0 | 0 | 0 | 0 | 0 | 0 | 0 | 0 | 0 | 0 | 0 | 0 | 0 | 0.00 | 0 |
| 16 | 0 | 0 | 0 | 0 | 0 | 0 | 0 | 0 | 0 | 0 | 0 | 0 | 0 | 0.00 | 0 |
| 18 | 0 | 0 | 0 | 0 | 0 | 0 | 0 | 0 | 0 | 0 | 0 | 0 | 0 | 0.00 | 0 |
| 27 | 0 | 0 | 0 | 0 | 0 | 0 | 0 | 0 | 0 | 0 | 0 | 0 | 0 | 0.00 | 0 |
| 28 | 0 | 0 | 0 | 0 | 0 | 0 | 0 | 0 | 0 | 0 | 0 | 0 | 0 | 0.00 | 0 |
| 29 | 0 | 0 | 0 | 0 | 0 | 0 | 0 | 0 | 0 | 0 | 0 | 0 | 0 | 0.00 | 0 |
| 31 | 0 | 0 | 0 | 0 | 0 | 0 | 0 | 0 | 0 | 0 | 0 | 0 | 0 | 0.00 | 0 |
| 33 | 0 | 0 | 0 | 0 | 0 | 0 | 0 | 0 | 0 | 0 | 0 | 0 | 0 | 0.00 | 0 |
| 40 | 0 | 0 | 0 | 0 | 0 | 0 | 0 | 0 | 0 | 0 | 0 | 0 | 0 | 0.00 | 0 |
| 43 | 0 | 0 | 0 | 0 | 0 | 0 | 0 | 0 | 0 | 0 | 0 | 0 | 0 | 0.00 | 0 |
| 44 | 0 | 0 | 0 | 0 | 0 | 0 | 0 | 0 | 0 | 0 | 0 | 0 | 0 | 0.00 | 0 |
| 48 | 0 | 0 | 0 | 0 | 0 | 0 | 0 | 0 | 0 | 0 | 0 | 0 | 0 | 0.00 | 0 |
| 53 | 0 | 0 | 0 | 0 | 0 | 0 | 0 | 0 | 0 | 0 | 0 | 0 | 0 | 0.00 | 0 |
| 56 | 0 | 0 | 0 | 0 | 0 | 0 | 0 | 0 | 0 | 0 | 0 | 0 | 0 | 0.00 | 0 |
| 60 | 0 | 0 | 0 | 0 | 0 | 0 | 0 | 0 | 0 | 0 | 0 | 0 | 0 | 0.00 | 0 |
| 61 | 0 | 0 | 0 | 0 | 0 | 0 | 0 | 0 | 0 | 0 | 0 | 0 | 0 | 0.00 | 0 |
| 63 | 0 | 0 | 0 | 0 | 0 | 0 | 0 | 0 | 0 | 0 | 0 | 0 | 0 | 0.00 | 0 |
| 68 | 0 | 0 | 0 | 0 | 0 | 0 | 0 | 0 | 0 | 0 | 0 | 0 | 0 | 0.00 | 0 |
| 82 | 0 | 0 | 0 | 0 | 0 | 0 | 0 | 0 | 0 | 0 | 0 | 0 | 0 | 0.00 | 0 |
| 84 | 0 | 0 | 0 | 0 | 0 | 0 | 0 | 0 | 0 | 0 | 0 | 0 | 0 | 0.00 | 0 |
| 88 | 0 | 0 | 0 | 0 | 0 | 0 | 0 | 0 | 0 | 0 | 0 | 0 | 0 | 0.00 | 0 |
| 89 | 0 | 0 | 0 | 0 | 0 | 0 | 0 | 0 | 0 | 0 | 0 | 0 | 0 | 0.00 | 0 |
| 91 | 0 | 0 | 0 | 0 | 0 | 0 | 0 | 0 | 0 | 0 | 0 | 0 | 0 | 0.00 | 0 |
| 94 | 0 | 0 | 0 | 0 | 0 | 0 | 0 | 0 | 0 | 0 | 0 | 0 | 0 | 0.00 | 0 |
| 97 | 0 | 0 | 0 | 0 | 0 | 0 | 0 | 0 | 0 | 0 | 0 | 0 | 0 | 0.00 | 0 |
| 99 | 0 | 0 | 0 | 0 | 0 | 0 | 0 | 0 | 0 | 0 | 0 | 0 | 0 | 0.00 | 0 |
| 104 | 0 | 0 | 0 | 0 | 0 | 0 | 0 | 0 | 0 | 0 | 0 | 0 | 0 | 0.00 | 0 |
| 108 | 0 | 0 | 0 | 0 | 0 | 0 | 0 | 0 | 0 | 0 | 0 | 0 | 0 | 0.00 | 0 |
| 109 | 0 | 0 | 0 | 0 | 0 | 0 | 0 | 0 | 0 | 0 | 0 | 0 | 0 | 0.00 | 0 |
| 111 | 0 | 0 | 0 | 0 | 0 | 0 | 0 | 0 | 0 | 0 | 0 | 0 | 0 | 0.00 | 0 |
| 114 | 0 | 0 | 0 | 0 | 0 | 0 | 0 | 0 | 0 | 0 | 0 | 0 | 0 | 0.00 | 0 |
| 115 | 0 | 0 | 0 | 0 | 0 | 0 | 0 | 0 | 0 | 0 | 0 | 0 | 0 | 0.00 | 0 |
| 120 | 0 | 0 | 0 | 0 | 0 | 0 | 0 | 0 | 0 | 0 | 0 | 0 | 0 | 0.00 | 0 |
| 121 | 0 | 0 | 0 | 0 | 0 | 0 | 0 | 0 | 0 | 0 | 0 | 0 | 0 | 0.00 | 0 |
| 130 | 0 | 0 | 0 | 0 | 0 | 0 | 0 | 0 | 0 | 0 | 0 | 0 | 0 | 0.00 | 0 |
| 134 | 0 | 0 | 0 | 0 | 0 | 0 | 0 | 0 | 0 | 0 | 0 | 0 | 0 | 0.00 | 0 |
| 136 | 0 | 0 | 0 | 0 | 0 | 0 | 0 | 0 | 0 | 0 | 0 | 0 | 0 | 0.00 | 0 |
| 137 | 0 | 0 | 0 | 0 | 0 | 0 | 0 | 0 | 0 | 0 | 0 | 0 | 0 | 0.00 | 0 |
| 141 | 0 | 0 | 0 | 0 | 0 | 0 | 0 | 0 | 0 | 0 | 0 | 0 | 0 | 0.00 | 0 |
| 142 | 0 | 0 | 0 | 0 | 0 | 0 | 0 | 0 | 0 | 0 | 0 | 0 | 0 | 0.00 | 0 |
| 143 | 0 | 0 | 0 | 0 | 0 | 0 | 0 | 0 | 0 | 0 | 0 | 0 | 0 | 0.00 | 0 |
| 145 | 0 | 0 | 0 | 0 | 0 | 0 | 0 | 0 | 0 | 0 | 0 | 0 | 0 | 0.00 | 0 |
| 146 | 0 | 0 | 0 | 0 | 0 | 0 | 0 | 0 | 0 | 0 | 0 | 0 | 0 | 0.00 | 0 |
| 152 | 0 | 0 | 0 | 0 | 0 | 0 | 0 | 0 | 0 | 0 | 0 | 0 | 0 | 0.00 | 0 |
| 153 | 0 | 0 | 0 | 0 | 0 | 0 | 0 | 0 | 0 | 0 | 0 | 0 | 0 | 0.00 | 0 |
| 156 | 0 | 0 | 0 | 0 | 0 | 0 | 0 | 0 | 0 | 0 | 0 | 0 | 0 | 0.00 | 0 |
| 157 | 0 | 0 | 0 | 0 | 0 | 0 | 0 | 0 | 0 | 0 | 0 | 0 | 0 | 0.00 | 0 |
| 158 | 0 | 0 | 0 | 0 | 0 | 0 | 0 | 0 | 0 | 0 | 0 | 0 | 0 | 0.00 | 0 |
| 161 | 0 | 0 | 0 | 0 | 0 | 0 | 0 | 0 | 0 | 0 | 0 | 0 | 0 | 0.00 | 0 |
|  |  |  |  |  |  |  |  |  |  |  |  |  |  |  |  |
| Mean | 0.38 | 0.7 | 0.5 | 0.6 | 0.3 | 0.2 | 0 | 0.2 | 0 | 0 | 0.1 | 0.7 |  |  |  |
| Sum | 60 | 110 | 74 | 101 | 49 | 25 | 21 | 25 | 25 | 18 | 19 | 110 |  |  |  |

Table A2. Covariates used in equation (6). Comparison of the summary statistics of the mixed linear model with covariates of 1, 2 and 3 months before mosquito collection. * coefficient significant at 0.05 level.

| ***Lag 1 month*** |  |  |
| --- | --- | --- |
| **Covariates (scaled)** | **Coefficient** | **Standard errors** |
| Air temperature | 0.521* | 0.227 |
| Wet bulb temperature | -0.625* | 0.226 |
| Relative humidity | 0.243* | 0.098 |
|  |  |  |
| ***Lag 2 months*** |  |  |
| **Covariates (scaled)** | **Coefficient** | **Standard errors** |
| Air temperature | -0.014 | 0.247 |
| Wet bulb temperature | -0.060 | 0.242 |
| Relative humidity | 0.072 | 0.105 |
|  |  |  |
| ***Lag 3 months*** |  |  |
| **Covariates (scaled)** | **Coefficient** | **Standard errors** |
| Air temperature | 0.257 | 0.269 |
| Wet bulb temperature | -0.270 | 0.264 |
| Relative humidity | 0.198 | 0.114 |
|  |  |  |

Table A3. Moran curve parameters at each trap (last row for the general model).

| *Idtrap* | *λ_0_, field fertility* | *d* | α (degrees) |
| --- | --- | --- | --- |
| 3 | 1.000 | 0.461 | 47.452 |
| 4 | 1.000 | 0.473 | 47.848 |
| 5 | 1.000 | 0.540 | 50.431 |
| 6 | 1.000 | 0.493 | 50.631 |
| 8 | 1.873 | 1.266 | 105.256 |
| 10 | 1.000 | 0.515 | 55.163 |
| 13 | 1.000 | 0.463 | 47.641 |
| 14 | 1.000 | 0.457 | 46.830 |
| 15 | 1.000 | 0.482 | 48.286 |
| 17 | 0.917 | 0.244 | 25.185 |
| 19 | 1.092 | 0.690 | 64.068 |
| 20 | 0.929 | 0.432 | 41.866 |
| 21 | 1.000 | 0.414 | 41.470 |
| 22 | 1.000 | 0.412 | 41.198 |
| 23 | 1.000 | 0.551 | 54.104 |
| 24 | 1.029 | 0.637 | 56.742 |
| 25 | 1.015 | 0.566 | 53.755 |
| 26 | 1.000 | 0.457 | 46.840 |
| 30 | 1.189 | 0.782 | 75.291 |
| 32 | 0.933 | 0.446 | 42.456 |
| 34 | 1.000 | 0.491 | 48.224 |
| 35 | 0.929 | 0.428 | 41.348 |
| 36 | 0.941 | 0.591 | 56.520 |
| 37 | 1.000 | 0.456 | 46.793 |
| 38 | 1.000 | 0.486 | 49.655 |
| 39 | 1.238 | 0.649 | 61.033 |
| 41 | 0.929 | 0.422 | 41.390 |
| 42 | 1.000 | 0.558 | 57.554 |
| 45 | 1.000 | 0.465 | 47.924 |
| 46 | 1.381 | 0.804 | 71.867 |
| 47 | 1.000 | 0.474 | 49.142 |
| 49 | 1.000 | 1.026 | 90.862 |
| 50 | 1.000 | 0.484 | 48.529 |
| 51 | 0.917 | 0.244 | 25.185 |
| 52 | 1.306 | 0.822 | 76.925 |
| 54 | 1.000 | 0.777 | 72.747 |
| 57 | 1.000 | 0.497 | 47.090 |
| 58 | 1.000 | 0.456 | 46.793 |
| 59 | 0.929 | 0.458 | 45.858 |
| 62 | 1.000 | 0.414 | 41.357 |
| 64 | 1.000 | 0.631 | 58.761 |
| 65 | 1.034 | 0.558 | 50.360 |
| 66 | 1.000 | 0.608 | 58.353 |
| 67 | 1.000 | 0.491 | 49.453 |
| 69 | 1.000 | 0.456 | 46.793 |
| 70 | 1.125 | 0.813 | 70.033 |
| 71 | 1.239 | 0.834 | 78.801 |
| 72 | 1.025 | 0.956 | 92.372 |
| 73 | 1.000 | 0.355 | 33.657 |
| 74 | 1.071 | 0.544 | 53.290 |
| 75 | 1.000 | 0.459 | 47.118 |
| 76 | 1.383 | 0.963 | 84.801 |
| 77 | 1.000 | 0.467 | 42.802 |
| 78 | 1.198 | 0.654 | 66.007 |
| 79 | 1.000 | 0.574 | 57.219 |
| 80 | 1.000 | 0.478 | 49.727 |
| 81 | 1.148 | 0.730 | 64.476 |
| 83 | 1.025 | 0.579 | 48.687 |
| 85 | 1.331 | 0.727 | 71.190 |
| 86 | 1.161 | 0.610 | 62.575 |
| 87 | 1.000 | 0.516 | 50.673 |
| 90 | 1.783 | 0.994 | 87.444 |
| 92 | 1.842 | 0.984 | 86.875 |
| 93 | 1.000 | 0.414 | 41.356 |
| 95 | 1.000 | 0.456 | 46.781 |
| 96 | 1.077 | 0.606 | 63.590 |
| 98 | 1.182 | 0.836 | 99.484 |
| 100 | 1.000 | 0.464 | 47.893 |
| 101 | 1.000 | 0.469 | 48.501 |
| 102 | 1.000 | 0.500 | 50.733 |
| 103 | 1.067 | 0.701 | 72.159 |
| 105 | 0.980 | 0.828 | 78.371 |
| 106 | 1.000 | 0.782 | 81.486 |
| 107 | 1.071 | 0.623 | 63.432 |
| 110 | 1.000 | 0.458 | 47.048 |
| 112 | 1.000 | 0.497 | 50.222 |
| 113 | 1.000 | 0.479 | 49.963 |
| 116 | 1.000 | 0.544 | 54.439 |
| 117 | 1.000 | 0.548 | 60.147 |
| 118 | 1.000 | 0.405 | 40.301 |
| 119 | 1.000 | 0.465 | 46.102 |
| 122 | 1.000 | 0.497 | 49.065 |
| 123 | 1.000 | 0.605 | 58.992 |
| 124 | 1.215 | 0.632 | 61.034 |
| 125 | 1.000 | 0.443 | 45.102 |
| 126 | 1.000 | 0.495 | 47.888 |
| 127 | 1.000 | 0.474 | 49.144 |
| 128 | 1.000 | 0.498 | 48.293 |
| 129 | 1.079 | 0.905 | 52.019 |
| 131 | 1.571 | 0.943 | 84.750 |
| 132 | 1.062 | 0.506 | 48.672 |
| 133 | 0.923 | 0.349 | 34.071 |
| 135 | 1.474 | 1.997 | 121.792 |
| 138 | 1.114 | 0.972 | 83.941 |
| 139 | 1.154 | 0.772 | 87.059 |
| 140 | 1.182 | 0.836 | 99.484 |
| 144 | 1.000 | 0.476 | 48.228 |
| 147 | 1.076 | 0.642 | 63.393 |
| 148 | 1.059 | 0.719 | 73.096 |
| 149 | 1.062 | 0.536 | 52.543 |
| 150 | 1.384 | 0.720 | 66.359 |
| 151 | 1.054 | 0.760 | 66.147 |
| 154 | 1.000 | 0.440 | 44.666 |
| 160 | 1.024 | 1.550 | 113.296 |
| General model | 1.048 | 0.585 | 56.281 |

Table A4. Summary statistics for the negative binomial generalized linear model fitting the mosquito densities at time t+1 (Nt1). * coefficient significant at 0.05 level.

| ***Fixed effect*** |  |  |
| --- | --- | --- |
| **Covariates** | **Coefficient** | **Standard errors** |
| Intercept | -1.176* | 0.111 |
| Nt (abundance) | -0.971* | 0.046 |
| Air temperature (scaled) | 0.135 | 1.559 |
| Wet bulb temperature (scaled) | -0.563 | 1.552 |
| Relative humidity (scaled) | 0.229 | 0.665 |
|  |  |  |
| ***Random effects*** | **Variance** | **Standard deviation** |
| Trap ID | 0.699 | 0.836 |

| **IGR** | **Real rho** | **Permuted**  **rho min** | **Permuted**  **rho max** | **Probability permuted rho** |
| --- | --- | --- | --- | --- |
| **Lag 0** |  |  |  |  |
| **Exact points** |  |  |  |  |
| Intrinsic growth | 0.89 | -0.70 | 0.95 | 0.003 |
| Abundance | 0.89 | -0.40 | 0.97 | 0.057 |
|  |  |  |  |  |
| **Lag 1** |  |  |  |  |
| **Exact points** |  |  |  |  |
| Intrinsic growth | 0.89 | -0.30 | 0.89 | 0.000 |
| Abundance | 0.89 | 0.60 | 1.00 | 0.720 |
|  |  |  |  |  |
| **Lag 2** |  |  |  |  |
| **Exact points** |  |  |  |  |
| Intrinsic growth | 0.89 | 0.05 | 1.00 | 0.580 |
| Abundance | 0.89 | 0.30 | 1.00 | 0.080 |
|  |  |  |  |  |
| **Lag 3** |  |  |  |  |
| **Exact points** |  |  |  |  |
| Intrinsic growth | 0.89 | 0.50 | 1.00 | 0.870 |
| Abundance | 0.89 | 0.30 | 1.00 | 0.130 |
|  |  |  |  |  |

## Appendix B. Supplementary Figure


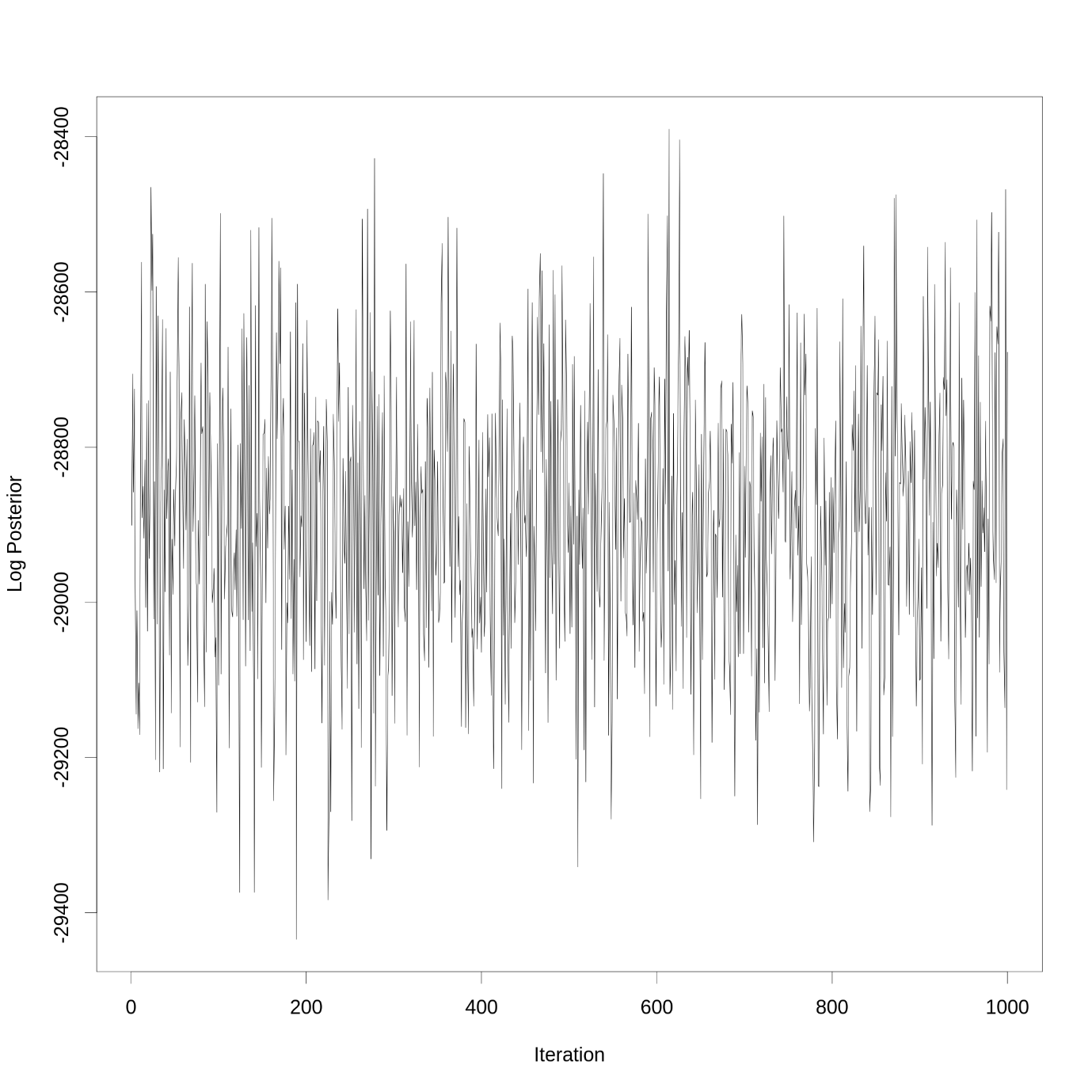


Figure B1. Global convergence plot for the parameters of the spatio-temporal log-Gaussian Cox model of dengue incidence with intrinsic growth rate as covariate.
